# *Drosophila melanogaster* Natural Variation Affects Growth Dynamics of Infecting *Listeria monocytogenes*

**DOI:** 10.1534/g3.115.022558

**Published:** 2015-10-04

**Authors:** Alejandra Guzmán Hotson, David S. Schneider

**Affiliations:** Department of Microbiology and Immunology, Stanford University, California 94305-5124

**Keywords:** Host-variation, bacterial growth dynamics, genetics of immunity

## Abstract

We find that in a *Listeria monocytogenes*/*Drosophila melanogaster* infection model, *L. monocytogenes* grows according to logistic kinetics, which means we can measure both a maximal growth rate and growth plateau for the microbe. Genetic variation of the host affects both of the pathogen growth parameters, and they can vary independently. Because growth rates and ceilings both correlate with host survival, both properties could drive evolution of the host. We find that growth rates and ceilings are sensitive to the initial infectious dose in a host genotype–dependent manner, implying that experimental results differ as we change the original challenge dose within a single strain of host.

When allowed to grow free in medium, bacteria grow in a logistic fashion; they can undergo a lag phase with little to no growth, then they grow exponentially until the environmental capacity limits growth, at which point the bacteria reach stationary phase (Monod 1949; Zwietering *et al.* 1990; Jemielita *et al.* 2014). Bacterial growth curves can thus be fit by a sigmoid defined by inoculum, maximal growth rate (r) and growth plateau (K) (Murray 2001). This description of logistic growth is a common starting assumption with many mathematical models that describe host–microbe interactions in hosts (for examples, see Anderson and May 1991; Nowak and May R 2000). Though growth within a host will obviously be more complicated than growth within medium because the host raises an immune response, we started with a simple logistic description as an entry-level model to monitor the growth of a model pathogen in the fly and to determine how genetic variation in the host altered the parameters of the model. Our logic was that once we developed an initial model we could add parameters to account for unexplained variance.

*Drosophila melanogaster* has three basic immune responses used to fight pathogens in the body cavity. First, the fly has a humoral response, which releases antimicrobial peptides (AMPs) into the circulation following activation of the Toll or IMD signal-transduction pathways in response to microbial products (Brennan and Anderson 2004). Second is the melanization response, in which the fly encapsulates and kills invaders through melanin deposition and the release of reactive oxygen species (Nappi *et al.* 2009). The third requires phagocytic cells, which engulf and digest pathogens (Kocks *et al.* 2005; Ulvila *et al.* 2011).

*Listeria monocytogenes* has been used as a tool to dissect immune function in the fly, much in the same way it has served as a tool in murine studies (Mansfield *et al.* 2003; Lara-Tejero and Pamer 2004; Hamon *et al.* 2006; Cossart and Toledo-Arana 2008; Dionne and Schneider 2008; Condotta *et al.* 2012). Injection of *L. monocytogenes* into the fly’s circulation causes a lethal infection. Infected flies lacking either the AMP or melanization response die more rapidly and contain more *L. monocytogenes* than do wild type flies (Mansfield *et al.* 2003; Ayres and Schneider 2008). The role phagocytes play in this infection is complicated because *L. monocytogenes* survives within these cells by escaping the phagosome and reproducing in the cytosol (Campbell 1994); an increase in phagocyte numbers increases the number of intracellular bacteria and decreases host survival (Chambers *et al.* 2012). We chose to study *L. monocytogenes* to measure the growth of a representative pathogen within the fly for three reasons: 1) it is simple to introduce consistent doses into the fly, 2) it is simple to measure growth, and 3) we already know that the fly varies in its response to *L. monocytogenes* in both resistance and tolerance (Ayres *et al.* 2008); thus this microbe seemed like a useful tool to probe variation in host immunity.

Natural variation affects immunity, and in *D. melanogaster* changes in bacterial growth properties are associated with natural variation of immunity genes (Lazzaro *et al.* 2004, 2006; Felix *et al.* 2012; Howick and Lazzaro 2014; Unckless *et al.* 2015). The effect of the fly genotype on median bacterial load is dependent on the bacterial pathogen such that a given host genotype does not have a universal effect on a range of bacterial pathogens (Lazzaro *et al.* 2006). Collections of genetically diverse *D. melanogaster* lines have been compiled to exploit natural genetic diversity to find the determinants of complex traits. One such source is the Drosophila Genomic Reference Panel (DGRP), a collection of 205 homozygous naturally derived *D. melanogaster* lines with diverse nucleotide polymorphisms (Mackay *et al.* 2012; Zhang *et al.* 2014). This resource has been used to find genetic determinants of immunity, for example, the nature of *Drosophila* resistance for two natural *D. melanogaster* viruses (DCV and DMelSV) and two viruses from other insects (FHV and DAffSV) (Magwire *et al.* 2012). We used this host variation to test how host genetics change pathogen growth dynamics.

We leveraged genetic variation in the host, using both natural variants from the DGRP as well as known *D. melanogaster* mutants, to evaluate the phenotypic range of bacterial growth during an infection. By infecting *D. melanogaster* with a known initial dose of *L. monocytogenes*, and measuring bacterial growth over the course of infection, we found that both maximal growth rates and plateaus varied between fly lines. The rates of bacterial growth and growth plateau did not correlate with each other, though both correlated with host survival. We demonstrate there are at least two parameters that quantitatively describe microbe growth, and that measurements of bacterial burden at multiple time points are required to describe differences in bacterial growth kinetics between host strains.

## Materials and Methods

### Fly stocks and husbandry

Flies were maintained on standard dextrose fly medium (129.4 g dextrose, 7.4 g agar, 61.2 g corn meal, 32.4 g yeast, and 2.7 g tegosept per liter) at 25° with 65% humidity and 12-hr light/dark cycles. Shortly after eclosion, adult flies were collected into bottles containing dextrose fly medium. At least 24 hr post eclosion adult flies were anesthetized with carbon dioxide, males were sorted into groups of 20 and placed into vials containing standard dextrose fly medium. Experiments were performed on flies 5–7 days post eclosion. CG2247 *piggybac* allele (BL18050), Kenny *piggybac* allele (BL11044), parental strain w^1118^ for both piggybac lines (BL6326), RAL 359 (BL28179), RAL 787 (BL28231), RAL 375 (BL25188), RAL 309 (BL28166), RAL 73 (BL28131), RAL 59 (BL28129), RAL 382 (BL28189), RAL 136 (BL28142), RAL 508 (BL28205), RAL 732 (BL25203), and RAL 821 (BL28243) strains were obtained from the Bloomington Stock Center.

### Injection

Bacteria were injected into flies essentially as described previously (Mansfield *et al.* 2003; Brandt and Schneider 2007; Ayres *et al.* 2008; Ayres and Schneider 2009). Briefly, flies were anesthetized with carbon dioxide. A drawn glass needle carrying *L. monocytogenes* was used to pierce the cuticle on the ventro-lateral side of the abdomen. A picospritzer III was used to inject 50 nl of liquid into the fly. Bacteria were delivered at different concentrations to produce injections of approximately 10, 100, 1000, 10,000 or 100,000 colony forming units (CFU). Precise infecting doses were determined for each experiment by plating a subset of flies at time zero.

### Bacterial strains and culturing conditions

*Listeria monocytogenes* wild type (10403S) stock was stored at –80° in brain and heart infusion (BHI) broth containing 25% glycerol. To prepare *L. monocytogenes* for injection, bacteria were streaked onto Luria Bertani (LB) agar plates containing 100 μg/ml streptomycin and incubated at 37° overnight. Single colonies of *Listeria* from the LB agar plate were used to inoculate 4 ml of BHI broth and incubated overnight at 37° without shaking. Bacteria were removed from the incubator at log growth phase and, prior to injection, *L. monocytogenes* cultures were diluted to the desired optical density (OD) 600 in phosphate buffered saline (PBS) and stored on ice.

### Bacterial plating

Single flies were homogenized in PBS using a motorized plastic pestle in 1.5 ml tubes. The supernatants were plated via spot-platting on LB agar plates containing 100 μg/ml streptomycin. When injecting 10 bacteria, flies were homogenized in 100 μl and all the PBS was plated for time points 0, 4, and 6 hr postinfection; beginning at 12 hr postinfection, flies were homogenized in 200 μl, and 100 μl was plated, followed by 10 μl spots of 1:10 serial dilutions (from the remaining 100 μl) in order to capture the full range of infection. When injecting 100 bacteria, flies were homogenized in 200 μl, 100 μl was plated, followed by 10 μl spots of 1:10 serial dilutions (from the remaining 100 μl) in order to capture the full range of infection. When injecting 1000 or more bacteria, flies were homogenized in 200 μl, and 10 μl spots of 1:10 serial dilutions were plated. At least eight samples were counted to determine the median number of bacteria for each inoculum. Plates were then incubated overnight at 37° before counting.

### Survival curve analysis

Sixty flies per line were injected and checked daily to measure mortality for each inoculum (PBS, 10^1^, 10^2^, 10^3^, 10^4^, 10^5^). Flies were housed in vials containing 20–25 flies each.

### Repetitions

Each set of conditions was repeated at least three times; for example, an experiment for a mutant fly line would be set up independently on three different days to gather microbe load and survival data.

### Curve fitting

The logistic curves were fit using Prism 6 software. The logistic equation used was: [Y = YM*Y0/((YM-Y0)*exp(–k*x) +Y0)], where “YM” is the growth plateau, “Y0” is the initial dose and “k” is the maximal growth rate. We transformed all the data using the natural log (LN) to obtain all of the constants and perform all regressions.

## Results

### Genetically diverse *Drosophila* host lines vary in their response to *L. monocytogenes*

**Figure 1:**
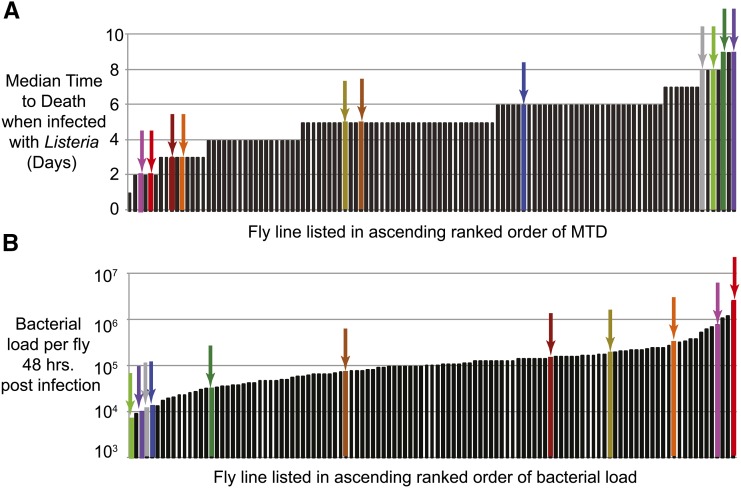
Median time to death (MTD) and bacterial load following *Listeria* infection vary in *Drosophila* naturally derived variants. Median value of (A) survival measured in median time to death (in days) and (B) bacterial load 48 hr postinfection measured in median colony forming units (CFU) when infected with 1000 CFU of *Listeria monocytogenes*. Arrows and colored columns represent each of the 11 RAL lines used for further study (colors are consistent throughout the figures): RAL 359 (light green), RAL 821 (purple), RAL 375 (dark yellow), RAL 59 (red), RAL 309 (gray), RAL 382 (pink), RAL 136 (dark green), RAL 508 (dark red) RAL 732 (brown), RAL 787 (blue), RAL 73 (orange).

To study host-associated variation in the parameters of pathogen growth, we first identified *D. melanogaster* lines with grossly different immune characteristics. We screened 114 lines from the DGRP (also referred to as the RAL lines) by injecting 1000 CFU of *L. monocytogenes* into 70 flies from each DGRP strain. We measured survival rates by recording median time to death (MTD), and measured bacterial loads at 48 hr postinfection by plating out homogenized flies. We found that these naturally varying lines exhibited a nine-fold range in MTD (from 1 to 9 days; Figure 1A and Supporting Information, Table S1) and an ∼350 fold range in bacterial load (from 6.44 × 10^3^ to 2.41 × 10^6^ CFUs; Figure 1B and Table S2). From these lines, we selected 11 for further study, choosing lines with a broad distribution of both survival rates and bacterial loads; these are not “the best” lines, rather they display a useful range of phenotypes (Figure 1 colored columns and arrows, and Table S3).

### Variation in *Listeria* growth dynamics in a *Drosophila* host can be explained using two host parameters

**Figure 2:**
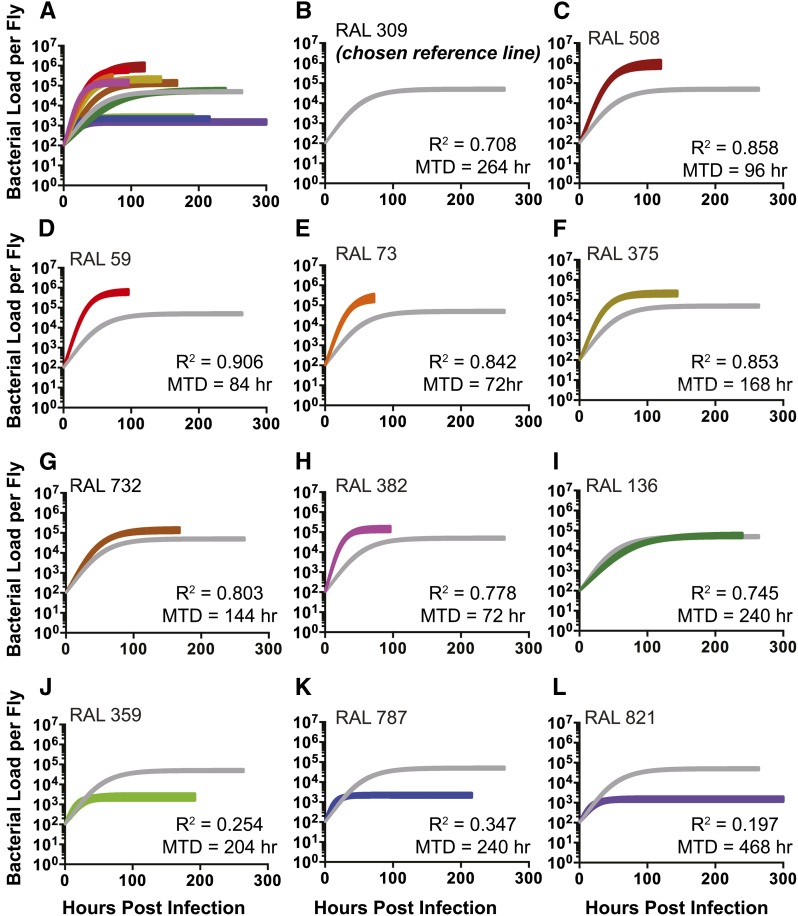
Logistic growth curves of Drosophila naturally derived variants differ primarily in the bacterial growth plateau. Microbial 11 growth curves showing the 95% confidence interval of 11 RAL-lines when infected with 100 L. monocytogenes. (A) All growth curves are on the same axis for comparison. We chose a reference line (B) RAL 309 and graphed the rest of the growth curves (C-L) as compared to RAL 309. The fit (R2) and survival (MTD in hr) for each curve is displayed. Bacterial load was LN transformed to fit logistic curve.

Our lab has shown that *L. monocytogenes* growth is grossly logistic in flies, and can be fit with three-parameter logistic curves that are defined by input load, maximal growth rate (r) and growth plateau (K) (A. Louie, personal communication). We anticipated that if growth of *L. monocytogenes* within the fly was really controlled by variation of r and K then we could observe this if we measured *L. monocytogenes* growth curves in these various fly lines. The logistic growth model predicts that when K is much larger than the input load of microbes, we would find that a logistic model provided a “good” fit for the data. However, as K dropped continuously to approach the input microbe load, bacterial growth would appear flat; under these conditions the variance in the system regarding microbe load would result in these low K lines having a less good fit than the high K. To investigate how these parameters might be affected by host genetics, we injected 100 CFU of *L. monocytogenes* into the 11 immune variant DGRP lines and followed the bacterial growth dynamics. We measured bacterial load at seven time points during the first 2 days of infection to capture the growth phase of the curve, and measured microbe loads daily until we reached the MTD for each fly line. The bacterial growth dynamics in the selected fly lines revealed variation in both maximal growth rate and growth plateau (Figure 2, Figure S1, Figure S2, Table 1 and Table S4). We measured bacterial growth plateaus that ranged over 550 fold (from 1.51 × 10^3^ to 8.51 × 10^5^). The bacterial loads from fly lines with bacterial growth plateau greater than or equal to 10^4^ CFU were fit well using a logistic curve (R^2^ > 0.7), as anticipated. We note that this is not a perfect fit and leaves room for further explanations to account for the 30% of variation that is not explained by the model. The logistic growth model fit the three lines with the lowest growth plateaus (ranging from around 1500 to 2300 CFU) less well (R^2^ between 0.20 and 0.35) than those with high K values. This is exactly what we anticipated with these lines as their K values are close to the input microbe load. An additional difficulty with these lines is that the variance we see in microbe growth exceeds the range of growth, which means that they are difficult to fit using any growth model (Figure S1 and Figure S2). A contributing factor to this problem is that microbe loads measured during stationary phase are the most variant; this hypothesis is supported by the improvement found in logistic fit when the curve is cut off at 72 hr postinfection instead of at MTD (R^2^ between 0.29 and 0.58; Table S5). Because of these problems, the calculated maximum growth rates for these three lines were imprecise, as seen by their large 95% confidence intervals (Figure S2 and Table 1). We surmise that it is difficult to measure the rates of microbe growth in these flies because the microbes undergo a small number of doublings.

**Table 1:** Bacterial growth parameters and survival to *Listeria* infection are significantly different among the natural varying fly lines

Name	K (Growth Plateau; LN Bacteria Per Fly)	K Standard Error	R (Maximal Growth Rate; LN Bacteria/ Hour)	R Standard Error	R^2^	MTD (hr)
RAL 508	13.66	±0.26	0.058	±0.0035	0.86	96
RAL 59	13.35	±0.16	0.07	±0.0032	0.91	84
RAL 73	12.4	±0.26	0.074	±0.005	0.84	72
RAL 375	12.36	±0.17	0.054	±0.0034	0.86	168
RAL 732	12.08	±0.18	0.036	±0.0024	0.8	144
RAL 382	11.87	±0.17	0.092	±0.006	0.78	72
RAL 136	11.24	±0.18	0.023	±0.0019	0.74	240
RAL 309	11.07	±0.08	0.028	±0.0013	0.72	264
RAL 359	7.75	±0.19	0.088	±0.022	0.25	204
RAL 787	7.7	±0.12	0.11	±0.02	0.35	240
RAL 821	7.32	±0.14	0.084	±0.019	0.2	468

The bacterial growth plateau and maximal growth rate (with the standard error) are listed here for all 11 of the RAL lines. The fit of the logistic curve (R^2^) and the MTD (hr) is also listed for each line. Data were LN transformed. LN, natural log; MTD, median time to death.

**Figure 3:**
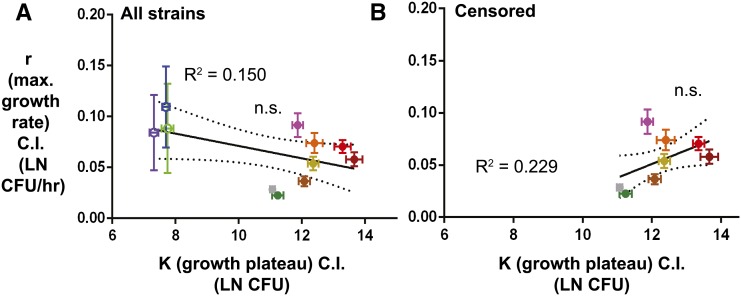
Maximal growth rate does not correlate with growth plateau. Correlating two microbial growth parameters, maximal growth rate and growth plateau, to each other, (A) using all 11 lines and (B) censoring the three lines with the lowest plateau and large 95% confidence intervals for maximal growth rate. Dashed lines represent the 95% confidence interval of the linear regression. n.s. signifies that the slope of the linear regression is not significantly different than zero. Bacterial load was LN transformed to get growth parameter measurements.

If growth rates and ceilings were tightly correlated, it might be reasonable to measure microbe loads at a single time point postinfection to understand growth dynamics. To test this idea, we measured the correlation between maximum growth rates and ceilings, and found no significant linear correlation between the parameters r and K. We repeated this analysis censoring the lines with low growth plateaus and still found no significant correlation (Figure 3). Negative results are always problematic, as one cannot conclude that there will never be a correlation between growth rates and plateaus, just that we did not observe a correlation in this study.

### *Listeria* growth rate and maximal loads both affect host health

**Figure 4:**
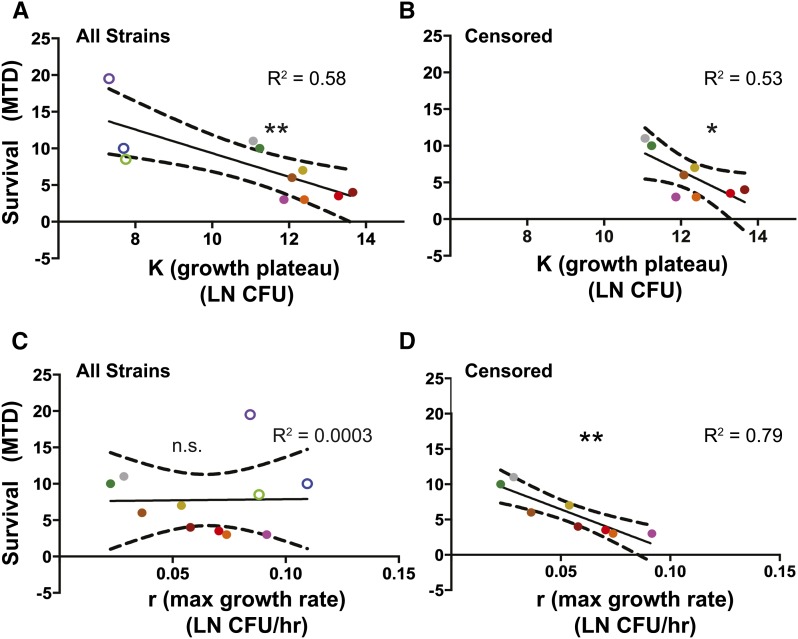
Survival negatively correlates with bacterial growth plateau and also with maximal growth rate when points with lowest K are removed: two microbial growth parameters are correlated with survival (MTD) as a measurement of health. The correlation with (A, C) all strains and (B, D) censored strains (without the three RAL lines with the lowest growth plateau and highest confidence intervals for the maximal growth rate). Growth plateau correlated with survival with all (A) and censored (B) data, while maximal growth rate is not correlated with survival with all (C) data, but it is with the censored (D) data. The 95% confidence interval is shown in the dashed-lines of the linear regression. Significance means the slope of the linear regression is significantly different than zero (*P < 0.05, **P < 0.01). Bacterial load was LN transformed to get bacterial parameter measurements.

It is important to understand not only how rapidly a pathogen grows, but also how that pathogen affects the health of the host; either the growth rate or the growth ceiling could impact host health. We used MTD as an indicator of host health and measured correlations of r and K with host MTD. We found the growth plateau to be linearly correlated with survival whether the three lines with the lowest plateaus were present or absent in the analysis (*P* < 0.01 and *P* = 0.04 respectively, Figure 4). In contrast, the maximal growth rate was not significantly linearly correlated with survival across all the fly lines used in this study, but there was a significant correlation if we excluded the three lines with the low growth plateau (*P* = 0.96 and *P* = 0.003, Figure 4). We felt it appropriate to censor the data from the three lines with the lowest plateaus, since the measurement of the maximal growth rate was imprecise because the effect size (growth) was smaller than the variance in microbe loads (Figure S2 and Table 1). A graph including the three lines with the low growth plateaus is not modeled well using a linear regression (R^2^ = 3 × 10^−4^), while the data does fit a linear regression when these points are absent (R^2^ = 0.793).

### The effect of infectious dose changes with different host genotypes

**Figure 5:**
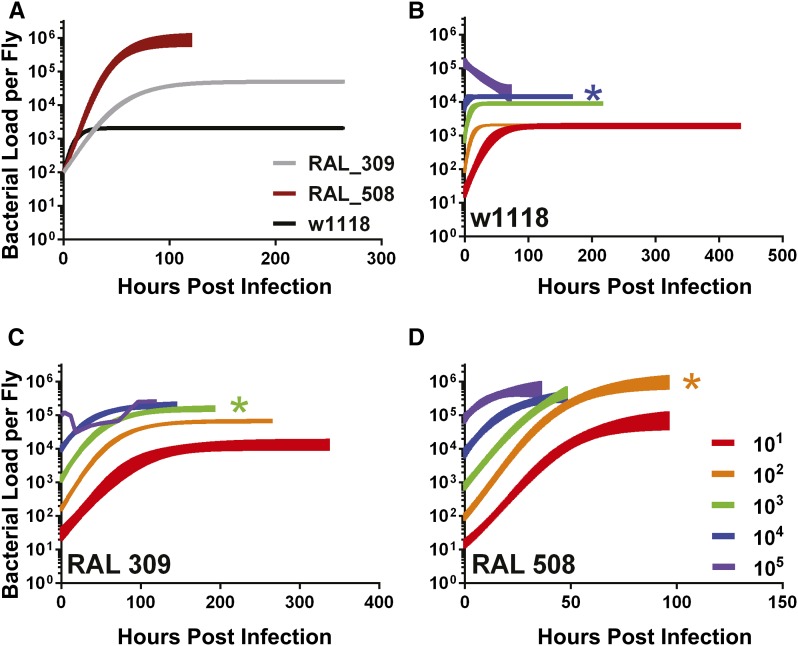
The effect of initial infectious dose on bacterial growth curve depends on host genetics: Microbial growth curve with different initial doses (10^1^, 10^2^, 10^3^, 10^4^, 10^5^) of three *Drosophila* lines. (A) Lines were shown to have different growth dynamics with an initial dose of 10^2^. The growth dynamics with different inocula of three lines: (B) w^1118^, (C) RAL 309, and (D) RAL 508. All curves were fit to a logistic curve and the 95% confidence interval is shown with the exception of RAL 309 with an initial dose of 10^5^ because the data did not fit a logistic curve; the median bacterial growth of each time point were connected instead. The three *Drosophila* genotypes have a different dose where the reach their maximal growth plateau (K_M_), which are shown with an asterisk (*) in the color of the first initial dose to reach the K_M_. Data were LN transformed to fit a logistic growth curve.

The above experiments were all performed with a single dose of *L. monocytogenes* but it is possible that growth rates and ceilings are dependent on input dose. We tested a commonly used “parental” line for many mutants, including those describe below, *D. melanogaster* strain w^1118^, along with two naturally derived strains, RAL 309 and RAL 508. These lines were selected in attempt to maximize the observed effects of infectious dose on growth dynamics, and to compare the growth dynamics of naturally derived fly lines with a well-characterized strain. When infected with 100 CFU, each of these three fly lines had different bacterial growth dynamics with respect to each other, with w^1118^ having a low growth plateau (Figure 2 and Figure 5A).

Each fly line was infected with a range of starting inocula (10^1^ – 10^5^ CFU). The growth plateau of the lowest inoculum was significantly lower than at the highest inoculum, indicating that the growth plateau of the system is dependent on the initial input. The dose-escalation of starting inocula revealed a maximal growth plateau (K_M_) achievable in each fly line; the lowest initial dose required to reach K_M_ was different for each line (10^2^ for RAL 508, 10^3^ for RAL 309, 10^4^ for w^1118^, Figure 5). We concluded that the effect of initial dose on the growth plateau depended on host genetics. For any given starting inoculum, the bacterial growth plateau was significantly different (*P* < 0.05) across the fly lines, with two exceptions: when infecting with 10^4^ CFU, the plateaus from the lines RAL 309 and RAL 508 were not significantly different, and when infecting w^1118^ with 10^5^ CFU the standard error was large and was not significantly different from the plateau from other two lines (Figure 5, Figure S3, Figure S4, Table 2 and Table S6). We found that the effect of initial dose on growth rate also depends on host genetics. For both naturally derived fly lines, the maximal growth rates did not significantly change across the range of tested starting infectious doses. However, w^1118^’s maximal growth rate when infected with 10 CFU was significantly lower than the maximal growth rate when infecting with a dose of either 10^2^ or 10^3^ CFU (Figure 5, Figure S3, Figure S4 and Table 2).

**Table 2:** Effect of initial dose of *Listeria* is significantly different among genotypically variable fly lines

Name	Initial Dose	K (Growth Plateau) (LN Bacteria Per Fly)	K 95% CI	R (Maximal Growth Rate) (LN Bacteria/ Hour)	R 95% CI	R^2^	MTD (hr)
w1118	10^1^	7.595	±0.11	0.05026	±0.0070	0.43	456
	10^2^	7.631	±0.053	0.1583	±0.014	0.52	312
	10^3^	9.107	±0.078	0.1752	±0.035	0.23	192
	10^4^	9.58	±0.086	0.2329	±0.21	0.022	144
	10^5^	5.896	±12.69	0.003272	±0.015	0.16	96
RAL 309	10^1^	9.564	±0.22	0.02526	±0.0034	0.49	312
	10^2^	11.14	±0.086	0.03044	±0.0016	0.73	264
	10^3^	11.98	±0.12	0.03172	±0.0027	0.7	192
	10^4^	12.23	±0.15	0.03405	±0.0051	0.55	144
	10^5^	12.45*	±0.48*	N/A	N/A	N/A	120
RAL 508	10^1^	11.39	±0.41	0.05702	±0.0050	0.78	96
	10^2^	13.9	±0.29	0.05573	±0.0035	0.86	96
	10^3^	14.57	±0.73	0.04879	±0.0064	0.79	48
	10^4^	12.61	±0.28	0.09029	±0.021	0.46	48
	10^5^	13.35	±0.39	0.1018	±0.044	0.26	36

The bacterial growth plateau and maximal growth rate (with the standard error) are listed here for different infectious doses of three different fly lines. The fit of the logistic curve (R^2^) and the MTD (hr) is also listed for each line. *RAL 309 with an initial dose of 10^5^ did not fit a logistic curve. The growth plateau was estimated by measuring the median maximal growth over the last two time points (using all replicates) and performing a Student t-test to find the standard error. Data were LN transformed. LN, natural log; MTD, median time to death N/A, not applicable.

### Mutations in defined “immunity genes” alter bacterial growth dynamics

**Figure 6:**
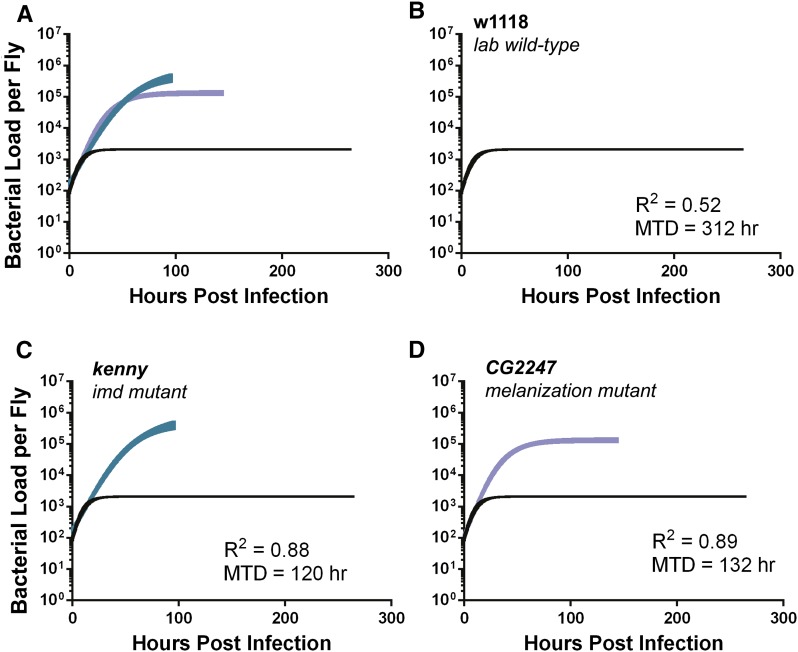
Logistic growth curves of *Drosophila* immune mutants differ primarily in the bacterial growth plateau. Microbial growth curves showing the 95% confidence interval of w^1118^ and two immune mutant lines when infected with 100 *L. monocytogenes*. (A) All growth curves are on the same axis for comparison, the rest are graphed with (B) w^1118^, the lab wild type. The two immune mutants are (C) *kenny* and (D) *CG2247*. The fit (R^2^) and survival (MTD in hours) for each curve is displayed. Bacterial load was LN transformed to fit logistic curve.

Having observed that natural variation across host genotypes has significant effects on bacterial growth dynamics, we next sought to understand how known immune mutations would affect bacterial growth dynamics. To test this, we infected the w^1118^ parental line and two *piggybac*-insertion mutant fly lines, *kenny*, a gene in the IMD signaling pathway, and the *CG2247*, a gene necessary for the melanization immune response (A. Louie, personal communication). Mutations in these two genes affected the dynamics of bacterial growth; the growth plateau of both mutants was significantly greater than the parental control (w^1118^), and the growth plateau of *kenny* was even higher than that of *CG2247* (plateau from *kenny* was 4.5-fold larger than that of *CG2247*, and *CG2247* was 60-fold larger than the plateau from w^1118^, *P* < 0.05). This increase in growth plateau was not accompanied by an increase in bacterial growth rate (Figure 6, Figure S5, Figure S6 and Table 3). To investigate the growth rates at a higher resolution we measured the bacterial levels hourly in all three lines for the first 12 hr of growth (Figure S7 and Figure S8). No significant difference in the exponential growth rate (Figure S7 and Figure S8) was observed between the three lines, indicating that the two immune mutant fly lines have an increased bacterial plateau but not a significantly larger maximal growth rate compared to the parental strain. Our evidence supports the hypothesis that the IMD pathway and melanization response reduce the *L. monocytogenes* growth plateau but does not support the hypothesis that this pathway alters *L. monocytogenes* growth rates.

**Table 3:** Bacterial growth parameters and survival to *Listeria* infection are significantly different between immune mutants and their parental line

Name	K (Growth Plateau; LN Bacteria Per Fly)	K Standard Error	r (Maximal Growth Rate; LN Bacteria/Hour)	r Standard Error	R^2^	MTD (hr)
Kenny	13.29	±0.26	0.041	±0.0024	0.87	120
CG2247	11.79	±0.11	0.064	±0029	0.89	132
w1118	7.64	±0.050	0.15	± 0.013	0.52	312

The bacterial growth plateau and maximal growth rate (with the standard error) are listed here for w^1118^ and two immune mutant lines. The fit of the logistic curve (R^2^) and the MTD (hr) is also listed for each line. Data were LN transformed. LN, natural log; MTD, median time to death.

## Discussion

We used a three-parameter model to describe the logistic growth of *L. monocytogenes* in *D. melanogaster*. We found that host genetics affects both maximal growth rate (r) and growth plateau (K) of *L. monocytogenes*, both of which correlate with a change in median time to death of the fly. We tested mutations in two immune pathways and found that this altered the growth plateau and not the maximal growth rate, thus melanization and IMD-induced responses affect the growth plateau but we have no evidence suggesting these processes affect *L. monocytogenes* growth rates. The effect of infectious dose changes in different host genotypes. There is a maximal growth plateau (K_M_) for each genotype, but the value of the K_M_ changes with host genotype.

The mathematical model we used to describe microbe growth is simple and explains more than 70% of the variation in microbe loads in fly lines that show more than three replication cycles of bacteria. This provides a step beyond past work in *Drosophila* where *L. monocytogenes* loads were measured at an arbitrary point post infection (Ayres *et al.* 2008; Ayres and Schneider 2008, 2009; Yano *et al.* 2008; Chambers *et al.* 2012a; Chambers and Schneider 2012); however, this logistic model is obviously incomplete because it ignores the immune response of the fly. To model the contribution of the immune response to *L. monocytogenes* growth, it will first be necessary to understand the nature of mathematical function describing immune induction as well as the parameters describing this function. To understand how growth and immunity affect host health a third equation will need to report the contribution of microbe numbers and the immune response on health. The work reported here provides a foundation for future studies that will use more sophisticated modeling approaches to measure the interactions between immune response and health of the host.

## Supplementary Material

Supporting Information
