# Supplementary material for: Drosophila melanogaster Natural Variation Affects Growth Dynamics of Infecting Listeria monocytogenes
Source: G3 (Bethesda). 2015 Oct 4;5(12):2593–600. doi: 10.1534/g3.115.022558 (PMC4683632; doi:10.1534/g3.115.022558)
Supplement: Supporting Information [file supp_5_12_2593__index.html]

Drosophila melanogaster Natural Variation Affects Growth Dynamics of Infecting Listeria monocytogenes — Drosophila melanogaster Natural Variation Affects Growth Dynamics of Infecting Listeria monocytogenes — Supporting Information 

# *Drosophila melanogaster* Natural Variation Affects Growth Dynamics of Infecting *Listeria monocytogenes*

## Supporting Information for Hotson and Schneider, 2015

**Files in this Data Supplement:**

- Table S1 - Median time to death of RAL-lines. (.pdf, 111 KB)
- Table S2 - Median percent of life shortened of RAL-lines. (.pdf, 28 KB)
- Table S3 - Immune phenotypes of RAL lines that are further studied. (.pdf,
- Table S4 - Parameters from logistic curves of analysis (one initial dose). (.pdf, 38 KB)
- Table S5 - Parameters from logistic curves of analysis (one initial dose). (.pdf, 38 KB)
- Table S6 - Statistical parameters from logistic curves analysis (varying initial dose). (.pdf, 40 KB)
- Figure S1 - .jpg, 137 KB
- Figure S2 - .jpg, 632 KB
- Figure S3 - .jpg, 782 KB
- Figure S4 - .jpg, 627 KB
- Figure S5 - .jpg, 656 KB
- Figure S6 - .jpg, 612 KB
- Figure S7 - .jpg, 668 KB
- Figure S8  - .jpg, 678 KB
