# Supplementary material for: Drosophila melanogaster Natural Variation Affects Growth Dynamics of Infecting Listeria monocytogenes
Source: G3 (Bethesda). 2015 Oct 4;5(12):2593–600. doi: 10.1534/g3.115.022558 (PMC4683632; doi:10.1534/g3.115.022558)
Supplement: Supporting Information [file supp_g3.115.022558_TableS1.pdf]

Table S1:

line	Injection MTD	line	Injection MTD	line	Injection MTD
RAL_105	4	RAL_378	7	RAL_75	4
RAL_109	6	RAL_379	4	RAL_765	6
RAL_136	9	RAL_38	5	RAL_774	5
RAL_149	4	RAL_380	7	RAL_786	5
RAL_158	4	RAL_382	2	RAL_787	6
RAL_161	5	RAL_385	6	RAL_790	5
RAL_176	2	RAL_387	6	RAL_796	3
RAL_177	4	RAL_398	6	RAL_801	7
RAL_208	6	RAL_40	5	RAL_804	6
RAL_21	4	RAL_405	5	RAL_808	6
RAL_228	6	RAL_406	5	RAL_810	4
RAL_235	5	RAL_42	6	RAL_812	6
RAL_237	5	RAL_427	6	RAL_818	4
RAL_239	5	RAL_437	7	RAL_819	3
RAL_28	8	RAL_443	5	RAL_821	9
RAL_280	6	RAL_45	6	RAL_822	5
RAL_287	5	RAL_486	6	RAL_837	6
RAL_301	5	RAL_491	9	RAL_843	5
RAL_303	6	RAL_502	6	RAL_85	5
RAL_304	6	RAL_508	3	RAL_850	4
RAL_309	8	RAL_513	4	RAL_852	6
RAL_313	3	RAL_517	6	RAL_853	3
RAL_315	6	RAL_530	6	RAL_855	5
RAL_318	6	RAL_535	6	RAL_857	5
RAL_320	6	RAL_563	5	RAL_859	6
RAL_321	1	RAL_57	4	RAL_861	5
RAL_324	4	RAL_589	5	RAL_879	5
RAL_325	5	RAL_59	2	RAL_882	7
RAL_336	5	RAL_595	5	RAL_884	3
RAL_338	3	RAL_639	4	RAL_890	6
RAL_350	4	RAL_703	4	RAL_897	3
RAL_352	5	RAL_705	6	RAL_900	5
RAL_356	2	RAL_712	2	RAL_907	5
RAL_358	5	RAL_716	7	RAL_908	4
RAL_359	8	RAL_73	3	RAL_91	6
RAL_370	5	RAL_730	8	RAL_913	4
RAL_371	5	RAL_732	5		
RAL_373	6	RAL_737	5		
RAL_375	5	RAL_738	5		
RAL_377	7	RAL_748	5		

Table S1 Median time to death of RAL-lines: The median time to death in days from the RAL lines infected with 1000 CFU of *L. monocytogenes*.
