# Supplementary material for: Drosophila melanogaster Natural Variation Affects Growth Dynamics of Infecting Listeria monocytogenes
Source: G3 (Bethesda). 2015 Oct 4;5(12):2593–600. doi: 10.1534/g3.115.022558 (PMC4683632; doi:10.1534/g3.115.022558)
Supplement: Supporting Information [file supp_g3.115.022558_TableS2.pdf]

line	Log CFU	line	Log CFU	line	Log CFU
RAL_101	4.986718647	RAL_379	5.37942568	RAL_765	4.605305046
RAL_105	5.039373367	RAL_38	4.783691418	RAL_774	5.020498846
RAL_109	4.36162519	RAL_380	3.943988875	RAL_783	4.969881644
RAL_136	4.49966751	RAL_382	5.877083257	RAL_786	5.133245204
RAL_149	4.962126717	RAL_385	4.98811284	RAL_787	4.103803721
RAL_158	4.954641051	RAL_387	5.197280558	RAL_790	4.581494542
RAL_161	5.138850438	RAL_40	5.513883186	RAL_796	5.572290606
RAL_176	5.705093264	RAL_405	5.000434077	RAL_801	4.120573931
RAL_177	5.230328668	RAL_406	5.193124598	RAL_804	4.898999271
RAL_181	6.021169602	RAL_42	4.230734256	RAL_808	5.084576278
RAL_208	4.801512612	RAL_427	4.35560404	RAL_810	4.970114322
RAL_21	4.969002035	RAL_437	4.409459182	RAL_812	4.670709595
RAL_217	4.511370855	RAL_443	4.838849091	RAL_818	5.492061605
RAL_227	5.832555869	RAL_45	4.572871602	RAL_819	5.562292864
RAL_228	4.748536734	RAL_491	4.548389418	RAL_821	3.977952121
RAL_235	5.314920056	RAL_502	4.961421094	RAL_822	5.210853365
RAL_237	5.100370545	RAL_508	5.171726454	RAL_837	5.100370545
RAL_239	4.621176282	RAL_513	5.098643726	RAL_843	4.796574333
RAL_280	5.41987725	RAL_517	4.487138375	RAL_849	4.465382851
RAL_287	5.08695426	RAL_530	5.138302698	RAL_85	5.098643726
RAL_309	4.065166884	RAL_535	4.433769834	RAL_850	5.080987047
RAL_318	5.321894069	RAL_563	4.804480189	RAL_852	4.846337112
RAL_320	4.671172843	RAL_57	4.887054378	RAL_853	4.963551734
RAL_325	5.18180599	RAL_589	5.320146286	RAL_855	5.128722284
RAL_336	5.042575512	RAL_59	6.382864073	RAL_857	5.496929648
RAL_338	5.341406354	RAL_595	5.181843588	RAL_859	5.201397124
RAL_340	4.532117116	RAL_639	5.267171728	RAL_861	6.072433726
RAL_350	5.013858231	RAL_703	4.974511693	RAL_879	5.141449773
RAL_352	5.290034611	RAL_705	4.694605199	RAL_882	4.276461804
RAL_356	4.877946952	RAL_712	5.781755375	RAL_884	5.193124598
RAL_358	5.13433578	RAL_712	5.359835482	RAL_890	4.556302501
RAL_359	3.808885867	RAL_716	4.862727528	RAL_897	5.009875634
RAL_370	5.021189299	RAL_73	5.390345833	RAL_900	4.958802703
RAL_371	4.669316881	RAL_732	4.875916969	RAL_907	5.08278537
RAL_373	4.29666519	RAL_737	4.810904281	RAL_908	4.675778342
RAL_375	5.23299611	RAL_738	4.896526217	RAL_91	5.370017435
RAL_377	4.662757832	RAL_748	4.757396029	RAL_913	5.213517757
RAL_378	4.744292983	RAL_75	5.302954057		

Table S2 Median percent of life shortened of RAL-lines: The bacterial load after 48 hours from the RAL lines infected with 1000 CFU of *L. monocytogenes*.
