# Supplementary material for: Drosophila melanogaster Natural Variation Affects Growth Dynamics of Infecting Listeria monocytogenes
Source: G3 (Bethesda). 2015 Oct 4;5(12):2593–600. doi: 10.1534/g3.115.022558 (PMC4683632; doi:10.1534/g3.115.022558)
Supplement: Supporting Information [file supp_g3.115.022558_TableS3.pdf]

Name	CFU Day 2	MTD
RAL 382	753,500	2
RAL 59	2,414,700	2
RAL 73	246,000	3
RAL 508	149,000	3
RAL 375	171,000	5
RAL 732	75,100	5
RAL 787	12,700	6
RAL 309	11,620	8
RAL 359	6,440	8
RAL 136	31,600	9
RAL 821	9,510	9

Table S3 Immune phenotypes of RAL lines that are further studied: The bacterial load after 48 hours and median time to death in days from each of the 11 lines studied are listed here. Lines were chosen to have different immune phenotypes.
