# Supplementary material for: Drosophila melanogaster Natural Variation Affects Growth Dynamics of Infecting Listeria monocytogenes
Source: G3 (Bethesda). 2015 Oct 4;5(12):2593–600. doi: 10.1534/g3.115.022558 (PMC4683632; doi:10.1534/g3.115.022558)
Supplement: Supporting Information [file supp_g3.115.022558_TableS4.pdf]

Table S4:

	RAL 375	RAL 309	RAL 359	RAL 821	RAL 59	RAL 732	RAL 382
Logistic growth							
Best-fit values							
YM	12.36	11.07	7.751	7.318	13.35	12.08	11.87
Y0	4.925	5.156	4.435	4.518	4.596	5.089	4.594
k	0.05386	0.02846	0.08817	0.08433	0.07044	0.03643	0.09164
Std. Error							
YM	0.1651	0.0801	0.1938	0.1378	0.164	0.1799	0.1682
Y0	0.1364	0.07074	0.3051	0.2356	0.1055	0.123	0.1398
k	0.003431	0.001265	0.02224	0.01887	0.003211	0.00244	0.005953
95% Confidence Intervals							
YM	12.04 to 12.69	10.92 to 11.23	7.369 to 8.132	7.048 to 7.589	13.03 to 13.68	11.73 to 12.43	11.54 to 12.20
Y0	4.656 to 5.193	5.017 to 5.295	3.834 to 5.035	4.055 to 4.981	4.388 to 4.804	4.847 to 5.331	4.319 to 4.869
k	0.04710 to 0.06062	0.02598 to 0.03094	0.04438 to 0.1320	0.04726 to 0.1214	0.06412 to 0.07675	0.03163 to 0.04123	0.07994 to 0.1033
Goodness of Fit							
Degrees of Freedom	240	1492	264	462	297	344	378
R square	0.8559	0.717	0.2539	0.1969	0.9059	0.803	0.7778
Absolute Sum of Squares	323.5	3209	1186	1909	320.3	557.6	801.7
Sy.x	1.161	1.467	2.119	2.033	1.039	1.273	1.456
Number of points							
Analyzed	243	1495	267	465	300	347	381

	RAL 136	RAL 774	RAL 787	6326	Kenny	RAL 73	CG2247
Logistic growth							
Best-fit values							
YM	11.24	13.66	7.699	7.64	13.29	12.4	11.79
Y0	5.105	4.518	4.572	4.525	5.136	4.438	4.752
k	0.02258	0.05786	0.1095	0.1516	0.04105	0.07384	0.06361
Std. Error							
YM	0.177	0.2552	0.1198	0.04989	0.2637	0.2575	0.1107
Y0	0.1257	0.1386	0.1985	0.09267	0.09536	0.1287	0.08871
k	0.001876	0.003534	0.02026	0.01329	0.002396	0.005019	0.002877
95% Confidence Intervals							
YM	10.89 to 11.58	13.16 to 14.16	7.463 to 7.935	7.542 to 7.738	12.77 to 13.81	11.89 to 12.91	11.57 to 12.01
Y0	4.858 to 5.352	4.245 to 4.791	4.181 to 4.962	4.343 to 4.707	4.948 to 5.324	4.185 to 4.692	4.577 to 4.926
k	0.01889 to 0.02627	0.05090 to 0.06483	0.06964 to 0.1493	0.1255 to 0.1777	0.03633 to 0.04577	0.06395 to 0.08372	0.05795 to 0.06927
Goodness of Fit							
Degrees of Freedom	405	243	364	849	256	239	342
R square	0.7446	0.8582	0.3467	0.5201	0.8889	0.8433	0.8923
Absolute Sum of Squares	767.3	418.1	962.8	1029	229.9	361.2	316.3
Sy.x	1.376	1.312	1.626	1.101	0.9476	1.229	0.9617
Number of points							
Analyzed	408	246	367	852	259	242	345

Table S4 Parameters from logistic curves of analysis (one initial dose): The parameters of each logistic fit on the RAL lines, w^{1118} and immune mutants are shown here. Data is cut off at the median time to death of each line. All lines were LN transformed to fit logistic curve.
