# Supplementary material for: Drosophila melanogaster Natural Variation Affects Growth Dynamics of Infecting Listeria monocytogenes
Source: G3 (Bethesda). 2015 Oct 4;5(12):2593–600. doi: 10.1534/g3.115.022558 (PMC4683632; doi:10.1534/g3.115.022558)
Supplement: Supporting Information [file supp_g3.115.022558_TableS5.pdf]

Table S5:

	RAL 375	RAL 309	RAL 359	RAL 821	RAL 59	RAL 732	RAL 382
Logistic growth							
Best-fit values							
YM	11.76	8.985	7.91	7.636	13.35	10.24	11.9
Y0	4.817	4.6	4.461	4.552	4.595	4.765	4.599
k	0.06217	0.07217	0.08054	0.07037	0.0705	0.0608	0.09109
Std. Error							
YM	0.2304	0.08444	0.2914	0.2542	0.1734	0.2354	0.1751
Y0	0.1258	0.05699	0.2404	0.1993	0.1018	0.1306	0.1373
k	0.004542	0.003508	0.01884	0.01565	0.003224	0.005781	0.005915
95% Confidence Intervals							
YM	11.30 to 12.21	8.820 to 9.151	7.335 to 8.485	7.136 to 8.136	13.01 to 13.69	9.781 to 10.71	11.55 to 12.24
Y0	4.569 to 5.065	4.488 to 4.711	3.986 to 4.935	4.160 to 4.944	4.395 to 4.796	4.507 to 5.022	4.329 to 4.869
k	0.05322 to 0.07113	0.06529 to 0.07906	0.04337 to 0.1177	0.03958 to 0.1012	0.06416 to 0.07685	0.04942 to 0.07218	0.07945 to 0.1027
Goodness of Fit							
Degrees of Freedom	198	937	182	308	289	268	370
R square	0.8562	0.7406	0.3726	0.2877	0.9108	0.7286	0.7833
Absolute Sum of Squares	199.5	848.2	508.4	939.3	285.3	365.1	753.1
Sy.x	1.004	0.9515	1.671	1.746	0.9937	1.167	1.427
Number of points							
Analyzed	201	940	185	311	292	271	373

	RAL 136	RAL 774	RAL 787	6326	Kenny	RAL 73	CG2247
Logistic growth							
Best-fit values							
YM	10.99	13.66	7.53	7.685	12.16	12.4	11.04
Y0	4.894	4.522	4.546	4.529	5.008	4.438	4.632
k	0.02926	0.05771	0.1223	0.1482	0.05072	0.07384	0.07716
Std. Error							
YM	0.3249	0.3371	0.1237	0.06796	0.2631	0.2575	0.1433
Y0	0.1151	0.1381	0.1256	0.07711	0.09777	0.1287	0.08176
k	0.002751	0.003965	0.01711	0.01181	0.0034	0.005019	0.003922
95% Confidence Intervals							
YM	10.35 to 11.63	13.00 to 14.33	7.286 to 7.774	7.551 to 7.818	11.64 to 12.68	11.89 to 12.91	10.76 to 11.32
Y0	4.667 to 5.120	4.250 to 4.794	4.298 to 4.793	4.377 to 4.680	4.816 to 5.201	4.185 to 4.692	4.471 to 4.793
k	0.02384 to 0.03468	0.04990 to 0.06552	0.08862 to 0.1560	0.1250 to 0.1714	0.04402 to 0.05742	0.06395 to 0.08372	0.06944 to 0.08488
Goodness of Fit							
Degrees of Freedom	260	229	238	547	240	239	286
R square	0.7224	0.8502	0.5791	0.6527	0.8698	0.8433	0.8873
Absolute Sum of Squares	338.8	373.1	239.4	457.4	203	361.2	203.2
Sy.x	1.142	1.276	1.003	0.9145	0.9197	1.229	0.8429
Number of points							
Analyzed	263	232	241	550	243	242	289

Table S5 Parameters from logistic curves of analysis (one initial dose): The parameters of each logistic fit on the RAL lines, w^{1118} and immune mutants are shown here. Data is cut of 72 hours post infection. All lines were LN transformed to fit logistic curve.
