# Supplementary material for: Drosophila melanogaster Natural Variation Affects Growth Dynamics of Infecting Listeria monocytogenes
Source: G3 (Bethesda). 2015 Oct 4;5(12):2593–600. doi: 10.1534/g3.115.022558 (PMC4683632; doi:10.1534/g3.115.022558)
Supplement: Supporting Information [file supp_g3.115.022558_TableS6.pdf]

Table S6:

	RAL 309 10^1	RAL 309 10^2	RAL 309 10^3	RAL 309 10^4	RAL 309 10^5	6326 10^1	6326 10^2	6326 10^3
Logistic growth					Not converged			
Best-fit values								
YM	9.564	11.14	11.98	12.23		7.595	7.631	9.107
Y0	3.388	5.253	7.13	9.223		3.144	4.382	6.761
k	0.02526	0.03044	0.03172	0.03405		0.05026	0.1583	0.1752
Std. Error								
YM	0.2248	0.08557	0.1246	0.1477		0.1087	0.05296	0.07809
Y0	0.2362	0.08825	0.119	0.1256		0.2121	0.09739	0.1988
k	0.00337	0.00156	0.00267	0.00509		0.00705	0.01416	0.03529
95% Confidence Intervals								
YM	9.122 to 10.01	10.97 to 11.31	11.73 to 12.22	11.94 to 12.52		7.382 to 7.809	7.527 to 7.735	8.953 to 9.260
Y0	2.924 to 3.852	5.080 to 5.426	6.896 to 7.364	8.976 to 9.471		2.727 to 3.560	4.191 to 4.573	6.370 to 7.152
k	0.01864 to 0.03189	0.02738 to 0.03349	0.02648 to 0.03696	0.02403 to 0.04408		0.03642 to 0.06411	0.1305 to 0.1861	0.1058 to 0.2445
Goodness of Fit								
Degrees of Freedom	432	1102	425	281		515	855	444
R square	0.4848	0.7129	0.7037	0.5454		0.4345	0.5128	0.2328
Absolute Sum of Squares	2752	2263	583	266.9		1790	1182	762.6
Sy.x	2.524	1.433	1.171	0.9745		1.864	1.176	1.311
Number of points								
Analyzed	435	1105	428	284	261	518	858	447

	6326 10^4	6326 10^5	RAL 508 10^1	RAL 508 10^2	RAL 508 10^3	RAL 508 10^4	RAL 508 10^5
Logistic growth							
Best-fit values							
YM	9.58	5.896	11.39	13.9	14.57	12.61	13.35
Y0	8.998	11.86	2.562	4.552	6.656	9.214	11.3
k	0.2329	0.003272	0.05702	0.05573	0.04879	0.09029	0.1018
Std. Error							
YM	0.08623	12.69	0.4068	0.2944	0.7264	0.2758	0.386
Y0	0.2161	0.1882	0.1708	0.1357	0.1577	0.232	0.1933
k	0.214	0.01541	0.00502	0.003498	0.00641	0.02117	0.0436
95% Confidence Intervals							
YM	9.410 to 9.750	-19.12 to 30.92	10.58 to 12.19	13.32 to 14.48	13.14 to 16.00	12.06 to 13.15	12.58 to 14.11
Y0	8.573 to 9.424	11.49 to 12.23	2.226 to 2.899	4.285 to 4.820	6.345 to 6.967	8.756 to 9.671	10.92 to 11.68
k	-0.1882 to 0.6540	-0.02710 to 0.03364	0.04713 to 0.06690	0.04884 to 0.06262	0.03615 to 0.06142	0.04854 to 0.1320	0.01557 to 0.1880
Goodness of Fit							
Degrees of Freedom	300	211	217	237	193	195	136
R square	0.02145	0.161	0.7707	0.861	0.783	0.3943	0.2584
Absolute Sum of Squares	441	425.2	554.6	389	257.6	423.2	172.4
Sy.x	1.212	1.42	1.599	1.281	1.155	1.473	1.126
Number of points							
Analyzed	303	214	220	240	196	198	139

Table S6 Statistical parameters from logistic curves analysis (varying initial dose): The parameters of each logistic fit on the five initial doses given to the three *Drosophila* lines. Data is cut off at the median time to death of each line. All lines were LN transformed to fit logistic curve.
