# Supplementary figures and images for: Drosophila melanogaster Natural Variation Affects Growth Dynamics of Infecting Listeria monocytogenes
Source: G3 (Bethesda). 2015 Oct 4;5(12):2593–600. doi: 10.1534/g3.115.022558 (PMC4683632; doi:10.1534/g3.115.022558)

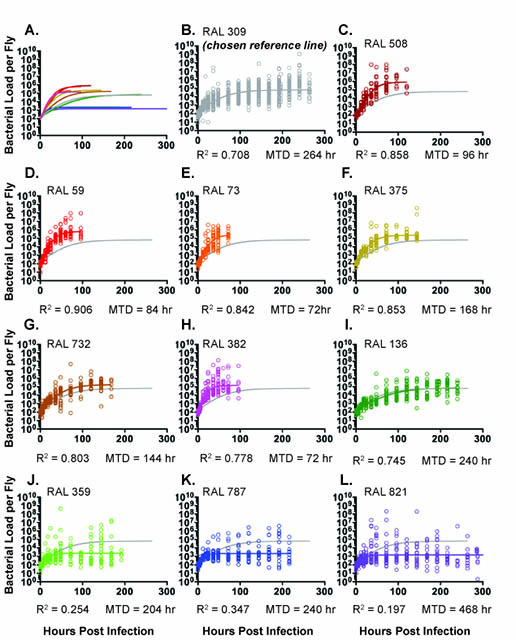

Supplement: Supporting Information [file supp_g3.115.022558_FigureS1.jpg]

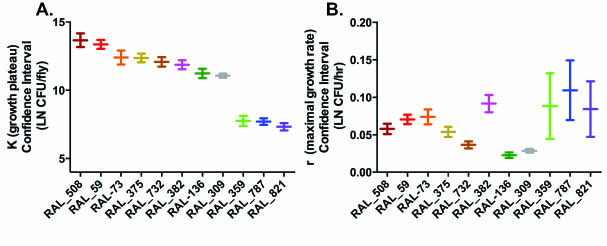

Supplement: Supporting Information [file supp_g3.115.022558_FigureS2.jpg]

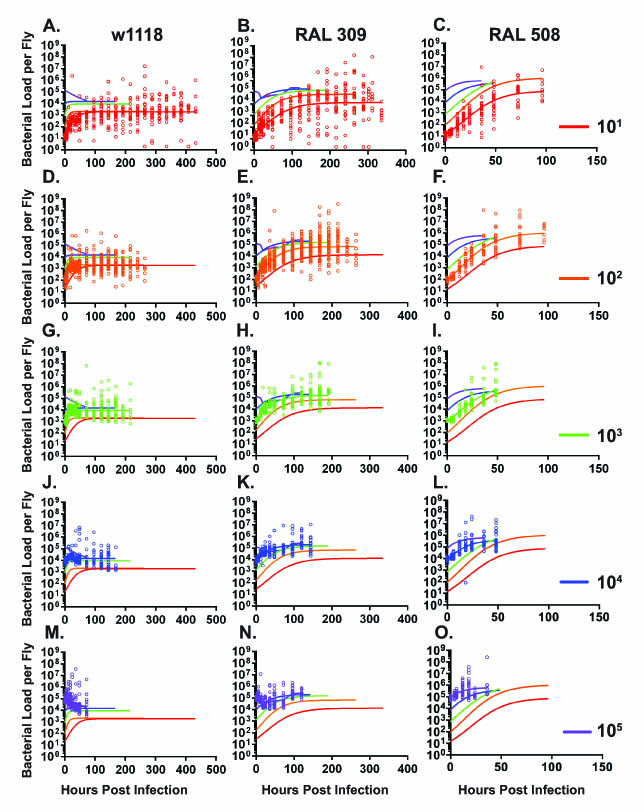

Supplement: Supporting Information [file supp_g3.115.022558_FigureS3.jpg]

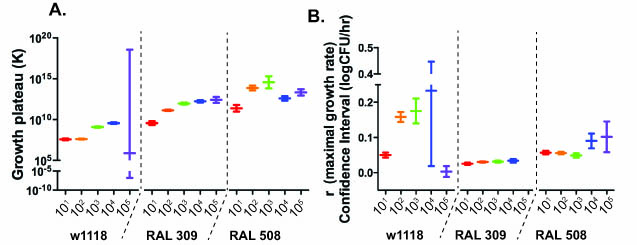

Supplement: Supporting Information [file supp_g3.115.022558_FigureS4.jpg]

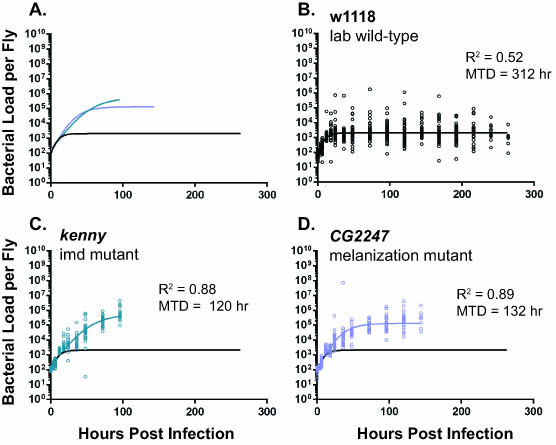

Supplement: Supporting Information [file supp_g3.115.022558_FigureS5.jpg]

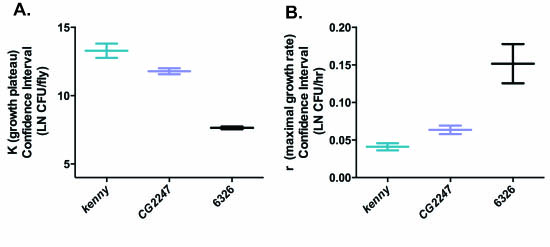

Supplement: Supporting Information [file supp_g3.115.022558_FigureS6.jpg]

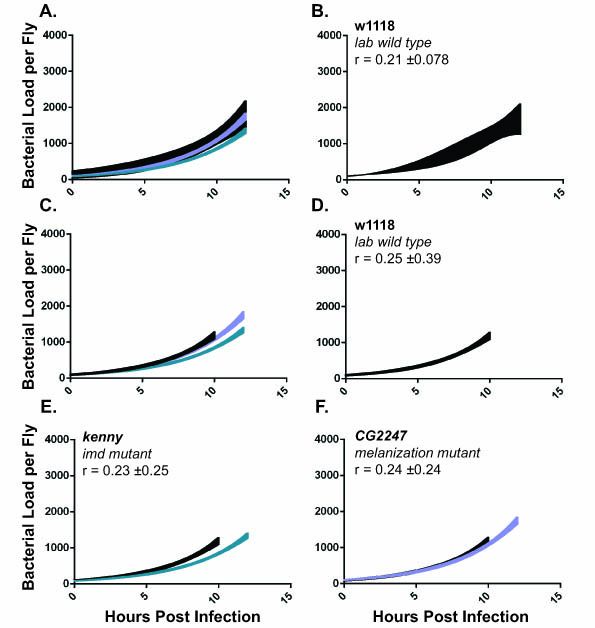

Supplement: Supporting Information [file supp_g3.115.022558_FigureS7.jpg]

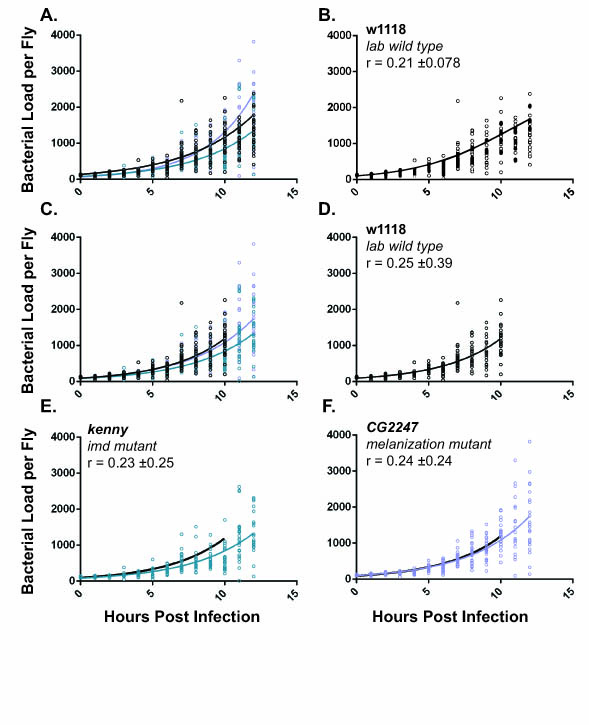

Supplement: Supporting Information [file supp_g3.115.022558_FigureS8.jpg]
